# Wound of the Intestine—Recovery

**Published:** 1867-01

**Authors:** 

**Affiliations:** Prairie du Chien, Wis.; Prairie du Chien, Wis.


### ARTICLE IV.

### WOUND OF THE INTESTINE—RECOVERY.

REPORTED BY DRS. MASON & WHITNEY, Prairie du Chien, Wis.

*Nov. 13th, 1866.* John Fisher, a native of Sweden, light complexion, tall, spare, and of robust constitution, *æt.* 32, was stabbed, in an affray, on the evening of the above day. The instrument by which the injury was inflicted was probably a large dirk-knife. The knife entered the abdominal cavity about an inch below the umbilicus, and nearly over the median line, producing a diagonal wound nearly two inches in length. The corresponding wound in the *transverse colon* was also diagonal, something over an inch in length, and was found to have divided a large branch of the mesenteric artery. When first examined, the larger portion of the intestines had escaped, including the wounded colon, and lay congested and cold upon the abdomen. There had been a good deal of hemorrhage from both ends of the severed artery, which was still bleeding. The pulse was feeble; surface cold and exsanguined; and the patient quite insensible.

A ligature was immediately placed on both ends of the divided artery and closely cut, the contents of the colon in the vicinity of the wound evacuated, the wound cleansed and closed accurately by the Glover's suture, care being taken to bring the serous surfaces in contact. The entire mass of intestines were now carefully examined, when, no other injury being found, they were cleansed with warm water and returned, the small intestines first, and, lastly, the wounded colon. The long ends of the intestinal suture were now drawn up, bringing with them the entire gut, so that the intestinal wound should lie directly under that of the abdomen, to which it was closely applied and secured by fastening the sutures outside. The external wound was now closed by two points of suture and a few straps of adhesive plaster, a pledget of dry lint and compress applied, and all secured by a broad bandage. The patient was ordered to have a little stimulus and ¼ grain of morphine every third hour.

*Nov. 14th.* Had passed a comfortable night; is well under the influence of morphia; pulse 108, quick; tongue a little red and dry; no tympanites, and very little pain. Ordered to continue the morphia, and, for nourishment, oatmeal gruel; for drink, ice-cold toast-water.

*Nov. 15th.* Pulse 120; tongue red; some tympanitis; lies quiet; narcotism pretty well established. Continue treatment.

*Nov. 16th.* Pulse 124; otherwise, same as yesterday; suffering no pain. Continue treatment.

*Nov. 17th.* Same as yesterday. Continue treatment.

*Nov. 18th.* Pulse 132, feeble; tongue very red and dry; tympanitis increased, and some pain. Continue treatment, and for nourishment, beef-tea.

*Nov. 19th.* Removed external sutures; found wound united, except at lower angle, where intestinal sutures were brought out; pulse 138; tympanitis about as yesterday. To increase the doses morphia to ½ gr. every four hours, and 1 oz. whiskey alternately.

*Nov. 20.* Pulse 132; tympanitis less; no pain. Continue treatment, and dress wound. Bowels moved a little to-day.

*Nov. 21st.* Pneumonia developed in the right lung; cough troublesome; some dyspnœa. Continue morphia, and substitute Mindererus' spirit for the whiskey.

*Nov. 22d.* Patient improved; pulse 124; surface perspiring; considerable rusty expectoration. Continue treatment.

*Nov. 23d, 24th, 25th, and 26th.* Improving; patient comfortable; intestinal suture coming away. Continue treatment.

*Nov. 28th.* Intestinal suture brought away; bowels moved freely. From this date, the patient recovered gradually, having a moderate diarrhœa only, which was restrained by tr. opii camph.

*Dec. 10th.* External wound about healed, and patient walking about.

It will be remarked that the morphia, pushed to moderate narcotism, was steadily persevered in throughout the treatment. Of course it was withdrawn as soon as the tympanitis began to subside and the danger from inflammation had passed. During the process of repair, a red and raised ring was noticed around the wound where, probably, the intestine was glued to the walls of the abdomen by plastic lymph.

